# On the Exposition of the Principles and Details of the Syllogism

**Published:** 1861-01

**Authors:** R. G. Latham


### Art. IV.—ON THE EXPOSITION OF THE PRINCIPLES AND DETAILS OF THE SYLLOGISM.

By R. G. Latham, M.D., F.R.S., &c.

In the present paper little or nothing will be said as to the utility of the so-called syllogistic logic; it being clear that the reasons in favour of, or against it, must lie less in the absolute value of the study itself, than in the relation borne to the time and trouble bestowed upon it. A comparatively unimportant pursuit that takes up but little time, and displaces but a small amount of other acquirements, may be a fitter object of education than a comparatively important one which demands the best energies of half a lifetime.

If so, the manner in which any subject is taught, is an important element in the value of the subject itself. Let the principles on which this is founded be bad, and let the details be irrelevant; let the unnecessary accessories be abundant, and let the doctrines of the different teachers be discordant, and the result will be, in the language of the great American utilitarian, *a whistle for which we pay more than it is worth.* That the whistle itself is a bad one, by no means follows. It may be worth much ; without being worth all we pay for it. Meanwhile, a whistle of inferior quality may be worth something—something which entails upon us no great sacrifice ; something in exchange for which even a whistle of moderate quality is a fair equivalent.

The present writer believes that if the elements of the so-called syllogistic logic were taught in an improved manner, they would be deemed worth learning by many who, at present, think meanly of them; such mild approbation as would be accorded being given on the principle that, if they were not, in any high degree, *bona per se,* they displaced but few of the numerous things which were better than themselves. That they have *some* value no one denies. They are important in the history of human thought; even if they be unimportant as rules for thinking. Let us (for argument's sake) put them at their lowest value; but (let us, as contributors to the art of education) divest them of all irrelevant details. The ratio between the price and the purchase will then come out.

In the order in which a subject is taken, we have important elements of either economy or waste. We waste power when a study is taken up without reference to what it leads to, and what leads to it. By taking it in its proper place we economize. This is more especially practicable when the higher departments of one, are the preliminaries to another branch of study. Reverse their order, and it is clear that the loss of time and the expenditure of attention will be considerable. Continue this reversal, or find imitators who will do it for you, and the result is a mischievous mass of detail improperly distributed. Preliminary matter of the kind just indicated, from having been omitted in its right place, has to be learnt in a wrong one. A little later, it becomes an inseparable adjunct, belonging, in appearance, to a field of inquiry, in which it is, in reality, a transplanted exotic. Later still, the boundaries of the field in which it stands become indistinct, and the field looks wider than it is. It also looks more impracticable, and, in the eyes of those who measure its fruits by its size, more barren.

Two subjects, between which a relation of the kind in question exists, are Language and Logic ; the preliminaries of the latter being found in the former—*when well and fully taught.* But as this is not the case, and as those who take up logic at all, for the most part take it up in ignorance of general grammar, a great deal that a schoolboy should have known from the Latin, Greek, or English accidence, has to form a sort of introductory chapter to logic—of which it soon passes for a part.

The sequence, however, of the several divisions of human knowledge, and the distribution of the details, belong to what we may call Mathematology in general. The question before us is the extent to which certain matters which (without belonging to Logic, appear to do so) are more properly relegated to Language, Psychology, and the Philosophy of the Classificational Sciences.

Everything connected with terms and copulas, with categorical and hypothetical propositions, with modes, with necessary and contingent matter, names, abstractions, generalizations, concepts, and the like, should be assumed as known *aliunde;* so that our subject should begin, not with the structure of propositions, but with their conversions, oppositions, and combinations.

Nevertheless, even in the present paper, it may not be unnecessary to state, as a preliminary, that—

The *Subject* is that which we speak about; the *Predicate*, that which we say concerning it. The *Copula* connects the two; and is either *is* or *is not.*

*Bread is cheap; bread is not cheap.*

Subjects and predicates are called terms; and where the two terms are connected by a copula we have a proposition. Propositions convey questions, commands and declarations, or statements. It is only the declaratory proposition that finds any place in logic.

Propositions, in respect to the *quality*, are either affirmative, as *this is mine;* or negative, as *this is not mine.* In respect to their *quantity*, they are either universal, as *all men are mortal;* or particular, as *some men are wise.*

By putting *quality* and *quantity* together we get four sorts of propositions, distinguished by the *signs* (1) all, (2) none, (3) some, (4) some-not; *e.g.*

1. *All men are mortal.*

2. *No man is immortal.*

3. *Some men are wise.*

4. *Some men are not wise.*

Of these

| | | |
|---|---|---|
| The first is an | Universal | Affirmative. |
| second | ,, | Negative. |
| third | Particular | Affirmative. |
| fourth | ,, | Negative. |

In the universal negative, the negation is pure and simple, and the sign, in the English language at least, is in direct contact with the subject. This is the case with *no man is perfect;* which, by transposition, becomes *no perfect being is a man.*

In the other, the negation, instead of being pure and simple, is accompanied by a second sign. This is, of course, the word *some*. The presence of this, in the English language at least, engenders a slight complication. Such an expression as—

*Some men are not heroes ;*

is not very easily transposed. We can only effect a transposition by connecting the negative with the predicate; as—

*Some not-heroes are men.*

This leads us to another question connected with what has just been exhibited ; viz., the transposition of terms. To say—

*Some men are fallible ;*

and—

*Some fallible beings are men ;*

is to say the same thing in different words; the two propositions being, to all intents and purposes, the same.

The difference between—

*No man is infallible ;*

and—

*No infallible being is man ;*

is verbal also.

With the universal affirmative it is otherwise. How often we hear such expressions as, *it does not follow that, because all men are two-legged, all two-legged animals are men ;* this being only one instance out of many. And what does it mean ? Simply that though

*All men are two-legged ;*

it is only

*Some two-legged animals that are men.*

That these two propositions are *really*, rather than *verbally*, different, is manifest. They exhibit a difference in extent between two classes of objects. The *men* belong to the smaller, the *two-legged animals* to the larger.

From what has preceded we may now see that the transposition or conversion of terms is pure and simple in the case of the particular affirmatives and the universal negatives only ; in each of which the predicate may become the subject, and the subject the predicate, by reversing the order of them.

How do the four kinds of propositions comport themselves towards each other? Knowing this, we have a logic of *two terms*.

*Every man is fallible ;*
*Some men are not fallible.*

Of these, one must be true, one false. To affirm the first is to contradict the second, and *vice versâ*. Propositions of this sort are called *contradictories*. They differ in both quality and quantity; one being affirmative and universal, the other negative and particular.

*Every man is fallible;*
*No man is fallible.*

Of these propositions each may possibly be false, and only one can, under any circumstances, be true. They are said to be *contrary*, or *contraries*, to one another. They differ in *quality;* one being affirmative, the other negative. In quantity they agree; both being universal.

*Some men are fallible;*
*Some men are not fallible.*

These are *subcontraries*. Both *may*, and one *must*, be true. They, like the ones by which they are preceded, differ in *quality*, but agree in *quantity*, being both particular. Only one, however, is affirmative.

*All men are fallible;*
*Some men are fallible.*

Here the truth of the universal, carries with, as a matter of necessity, the truth of the particular, proposition. If *each and all the* members of a given class have a certain quality, *some* of them *must* have it. Propositions of this kind are called *subaltern*. They differ in *quantity*, agreeing in *quality*. The ones under notice are affirmative.

*No man is perfect; some men are not perfect*, are negative. Still, the difference is limited to their quantities—one being universal, one particular.

Every term implies a class; *man*, that of *human beings; white*, that of *white objects*. Classes are of different magnitudes; some being larger, some smaller than others. The class of *heroes* is smaller than that of *men;* the class of *men* smaller than that of *mortal beings;* that of *mortal beings* smaller than that of *beings* in general. Strictly speaking, all this is extra-logical; belonging, in part, to general grammar, in part, to the sciences of classification. The term *subaltern*, however, is more logical than aught else; though the terms *genus* and *species* are not so. Now, with fear of being charged with making an unnecessary remark, we may remind the naturalist that, in the preceding series, the *beings*, at the one end, form a *genus;* whereas the *heroes*, at the other, form a *species*. The interjacent class is *genus* or *species* as the case may be. To the class indicated by *heroes*, the class indicated by *men*, is a *genus;* being itself but a *species,* to the class indicated by *mortals.* Such is subalternation.

And now we are on the logic of *three* (as opposed to the logic of *two*), terms—the logic of the *syllogism,* or the logic of *mediate* inference. What constitutes the logic of two terms, along with the consideration of the value of the division here suggested, may be considered hereafter. Our present object is to exhibit the doctrine of the ordinary syllogism in a simple form. It is this and something more. It is to show that its simplicity is great; its complexities and difficulties few. Here the exposition of its fundamental details begins. In a few pages, they will end. If many works on logic make even the elements difficult, the fault lies with the treatment of the subject rather than the subject itself.

The logic of the syllogism presumes the existence of a class —indeed, of three classes. Of the principle upon which these classes are formed it knows nothing. But it is clear that any individual, or any group of individuals which belongs to the smaller of them must also belong to the larger; must be included in it; even as the embryo contained in the kernel of a nut must needs be contained in the shell. If so, all that is wanted is the name of the individual or class. Get this, and you get a new name; get this, and you get the logic of *three* terms.

This brings in the terms *major* and *minor,* technical and logical terms. The predicate is, *essentially,* a major term; *i.e.,* the term indicating the *larger* class. The minor subject is, *essentially,* the minor term; *i.e.,* the term indicating the *smaller* class. That the major is *essentially* greater than the minor must be remembered. But it must also be remembered that the difference of magnitude is not always to be found. A *genus* is essentially larger than a *species;* but, if the genus consists of only a single species, the magnitudes of the two classes coincide. In no case, however, is the species *larger* than the genus. *Mutatis mutandis,* this applies to majors and minors. The minor is often the smaller, never the larger, class.

And now, after saying that *all heroes are men,* say that *all men are mortal.* In this case *heroes* is still a *minor,* and *men* a *major.* But it is not a *maximus.* Or say that *Cæsar* is a *hero.* In this case *hero* is still a *minor,* though not a *minimus.*

We do not, however, use the terms *maximus* and *minimus,* We call the intermediate term a *middle* term—the word being, again, technical. It is the name of a *subaltern* class. Now any member of a subaltern class must, perforce, be a member of the class to which the subaltern is subordinate; in other words, a minor which is in a middle *must*, also, be in a major. If *all men* (*middle*) *are mortal* and *all heroes* (*minor*) *are men, all heroes* MUST *be mortal.*

*All men are mortal ;*
*All heroes are men ;*
*All heroes are mortal.*

The same, *mutatis mutandis,* in cases of exclusion.

We may call all this a truism. Truism or not, it is the basis of a great part of logic.

The term determines the proposition ; *i.e.*, the proposition which contains the major term is the major proposition, whilst that which contains the minor is the minor. The term which contains the inference is called the conclusion. The three together constitute the syllogism : of which the first two are called the premises. One premise contains the major and middle, and the other the minor and the middle. Their order is indifferent. In the conclusion the minor term is the subject, the major the predicate.

Where the terms are capable of pure and simple transposition, or conversion, the difference between the predicate and the subject is abolished ; or rather the same term may be either one or the other. The same term, under similar circumstances, is also either a major or a minor ; or, rather there is no apparent difference between the two, the relative magnitudes of the two classes being incapable of being measured.

We may say—

1.

*No man is perfect ;*

or—

*No perfect being is man.*

2.

*Some men are wise ;*

or—

*Some wise beings are men.*

From two particular propositions in the ordinary syllogism, nothing can be inferred : neither can anything be inferred from two negatives. Let the major, however, be universal (either affirmative or negative), and let the minor (subject to the rule against the concurrence of two negatives) be anything whatever, and, of some sort or other, a conclusion *must* follow. Of this conclusion the nature is regulated by the premises. It cannot be more affirmative than the more negative, nor more general than the more particular, of the two. Thus with—

*No man is perfect ;*
*Some rational beings are men ;*

the inference is,

*Some rational beings are not perfect.*

The order of the *premises* (as aforesaid) is indifferent. Whether we say—

*All men are mortal;*
*Socrates is a man;*
*Socrates is mortal;*

or—

*Socrates is a man;*
*All men are mortal;*
*Socrates is mortal;*

is indifferent.

The order of terms, when they are reciprocally interchangeable, is indifferent. We can say—

*Some rational beings are mortal;*

or—

*Some mortal beings are rational;*

just as we can say—

*No man is perfect;*

or—

*No perfect being is a man.*

When the proposition, however, is universal and affirmative, no such conversion can take place.

*All men are mortal;*

is not equivalent to—

*All mortals are men.*

In common language, expressions, like *some men are wise*, imply that *some* are not. In logic, *some* merely means *more than none*. It *may* mean *one*; it *may* mean *all*.

Again, singular terms, like *Socrates*, &c., are universal. What we say of Socrates we say of the *whole of him*. Hence, such a proposition as—

*Cæsar is a hero;*

is as truly universal as such a one as

*All heroes are men.*

The preceding conditions give us a conclusion. Let us now add that none but them gives us one.

If so, we have only to calculate the combinations. Doing this, we begin with propositions considered in respect to their Quality and Quantity only.

1.

1. Universal affirmative.
2. Universal affirmative.
3. Universal affirmative.

2.

1. Universal negative.
2. Universal affirmative.
3. Universal negative.

3.

1. Universal affirmative.
2. Particular affirmative.
3. Particular affirmative.

4.

1. Universal negative.
2. Particular affirmative.
3. Particular negative.

5.

1. Universal affirmative.
2. Particular negative.
3. Particular negative.

EXAMPLES.

1.

1. *All men are fallible ;*
2. *All heroes are men ;*
3. *All heroes are fallible.*

2.

1. *No man is perfect ;*
2. *All heroes are men ;*
3. *No hero is perfect.*

3.

1. *All men are fallible ;*
2. *Some rational beings are men ;*
3. *Some rational beings are fallible.*

4.

1. *No man is perfect ;*
2. *Some rational beings are men ;*
3. *Some rational beings are not perfect.*

5.

1. *All slaves are discontented ;*
2. *Some Africans are not discontented ;*
3. *Some Africans are not slaves.*

The sequence of the propositions as universal affirmative, universal negative, and the like, gives to the syllogism what is called its *mood.* We have just seen that the moods are five in number—five in number, and no more.

The number of syllogisms, however, is greater than that of the moods. This is because there is such a thing as *figure.* Transpose, when it can be done, our terms, and we shall see what this means. We can tell, too, *à priori,* what transposition will do. In every negative, and in every particular proposition, it will give a fresh form.

1. No change ; both the premises being unsusceptible of conversion.

2. Conversion of—

1. *No man is perfect ;*
2. *All heroes are men ;*
3. *No hero is perfect.*

into—

1. *No perfect being is a man ;*
2. *All heroes are men ;*
3. *No hero is perfect.*

3. Conversion of—

1. *All men are fallible ;*
2. *Some rational beings are men ;*
3. *Some rational beings are fallible ;*

into—

1. *All men are fallible ;*
2. *Some men are rational beings ;*
3. *Some rational beings are fallible.*

4. From the fourth we get two varieties ; inasmuch as the major proposition is convertible because it is negative, the min r because it is particular. Thus—

1. *No man is perfect ;*
2. *Some rational beings are men ;*
3. *Some rational beings are not perfect :*

gives either—

1. *No perfect being is a man ;*
2. *Some rational beings are men ;*
3. *Some rational beings are not perfect ;*

or—

1. *No man is perfect;*
2. *Some men are rational;*
3. *Some rational beings are not perfect.*

5. Here we have, as a concurrent syllogism,

1. *All slaves are wronged;*
2. *Some slaves are not discontented;*
3. *Some wronged beings are not discontented.*

The place of the *middle term* gives us the *figure*. In the first figure it is the subject of the major, and the predicate of the minor premise. In the second it is the predicate, in the third the subject, of both.

The number, then, of *fundamental* syllogisms is ten. What is meant by *fundamental*?

Propositions are what is called *strong* or *weak*; their *strength* or *weakness* being determined by their quantity. The meaning of this lies so near the surface that the terms under notice can scarcely be called technical. They are just a little metaphorical. *All* is a *stronger* term than *some*; and *some* a *weaker* term than *all*. In like manner, *all men are mortal* is a stronger proposition than *some men*, &c. That strong terms may be weakened (by writing *some* instead of *all*) is clear.

In logic, as in mechanics, nothing is stronger than its weakest part; in other words, no conclusion can be more general than the most particular of its premises. A conclusion too strong for the premises, or a conclusion for which the premises are too weak, is no conclusion at all.

But what if the premises be too *strong*? They *may* be so; and that in more ways than one.

*Elephants are stronger than horses;*
*Horses are stronger than men;*

therefore

*Elephants must be stronger than men.*

This is true; yet the middle term is irregular.

Again—

*Some men are mortal;*
*All men are rational;*

gives

*Some rational beings are mortal,*

as a conclusion—a conclusion which is particular. That the first premiss would be strengthened by being changed into

*All men are mortal,*

is clear. Yet the conclusion would be the same. Write—

*All men are mortal;*
*All men are rational:*

and the conclusion that

*All mortals are rational,*

is as far off as ever. All that we can say is that *some* are. But this we have inferred already.

Premises, then, are *adequate* when they are neither too weak nor too strong for the conclusion. Where there is strength in excess, there is adequacy, and something more.

Where the premises are simply adequate, (neither more nor less), the syllogism is *fundamental,* the fundamental syllogisms being (as already stated), ten in number—ten in number, and no more. How many of these can be strengthened? Two. For—

*All men are fallible;*
*Some men are rational;*

we may write—

*All men are fallible;*
*All men are rational;*

and the conclusion will be the same; *i.e.*

*Some rational beings are fallible.*

And for—

*Some slaves are not discontented;*
*All slaves are wronged;*
*Some wronged beings are not discontented;*

we may write—

*No slave is discontented;*
*All slaves are wronged;*
*Some wronged beings are not discontented.*

The *fact,* of course, is less true than it was originally: the inference, however, is the same—except that it is deduced *à fortiori.*

And now let us go over the ten fundamental syllogisms again—

1.

*All men are fallible;*
*All heroes are men;*
*All heroes are fallible.*

In the first figure. Unsusceptible of variation.

2.

*No man is perfect;*
*All heroes are men;*
*No hero is perfect.*

In the first figure. One variation.

3.

*All men are fallible ;*
*Some rationals are men ;*
*Some rationals are fallible.*

In the first figure. One variation.

4.

*No man is perfect ;*
*Some rationals are men ;*
*Some rationals are not perfect.*

First figure. Two variations.

5.

*All slaves are discontented ;*
*Some Africans are not discontented ;*
*Some Africans are not slaves.*

Second figure.

6.

*Some slaves are not discontented ;*
*All slaves are wronged ;*
*Some wronged beings are not discontented.*

Third figure.

7.

*No perfect being is a man ;*
*All heroes are men ;*
*No hero is perfect.*

Third figure.

8.

*All men are fallible ;*
*Some men are rational ;*
*Some rational beings are fallible.*

Third figure.

9.

*No perfect being is a man ;*
*Some rational beings are men ;*
*Some rational beings are not perfect.*

Second figure.

10.

*No man is perfect ;*
*Some men are rational ;*
*Some rationals are not perfect.*

Third figure.

Now, although these syllogisms have been exhibited as if the first figure were the original from which the others were derived, we must guard against assuming this to be the exact fact. It is a fact that, just as the first figure can be converted into the second or third, the second or third can (by simply reversing the process) be converted into the first. It is also a fact that, in the first figure the differences of major, middle, and minor are the most decided. Thirdly, it is only the first figure that gives a universal conclusion. Hence, the first figure has been deemed the highest, noblest, and most typical. It may, or may not, be this. The error against which we have to guard ourselves is the notion, which the preceding details have a tendency to engender, that it is the first figure in which we *think*. We rarely think in any figure; nor does the logic of the syllogism pretend that we do. All that the logic of the syllogism asserts is, that when we think in three terms, and those terms give us a conclusion, our thought is reducible to one of the ten forms under notice.

That our thoughts, however, approach certain figures in some cases, and others in others, should be known. It is probable that the sequence,

*All heroes are men;*
*All men are mortal;*

is commoner than—

*All men are mortal;*
*All heroes are men;*

That—

*Aristides was virtuous;*
*Aristides was a pagan;*

is clearly more natural than—

*Aristides was virtuous;*

*Some pagan was Aristides.*

The generality of our expressions may be improved. All that has been hitherto exhibited has been exhibited by means of special and concrete examples. But logic is essentially a formal science. All it requires is, that the conclusion should be true to the premises, no matter whether the premises themselves be true or false. From two true facts we may draw a conclusion which is false; *i.e.*, no conclusion at all; from two false facts we may draw a conclusion that should be unexceptionable. Provided that the reasoning be good, the data may be good, bad, or indifferent. And this distinction is important, inasmuch as it is useful to know in a controversy what part of the argument you object to. That *all swans are black*, is bad zoology. Nevertheless, if we look to the reasoning only, the syllogism—*all swans are black;*

*all geese are swans; all geese are black*—is valid. On the contrary, that *some heroes are Europeans* is true, that *some Greeks are heroes* is true also; and equally true is it that *some Greeks are Europeans;* the reasoning, however, that *some heroes are Europeans; some Greeks are heroes; some Greeks are Europeans,* is no reasoning at all; nor do the three propositions constitute a syllogism. Though three truths, they are three separate ones, and, to use an old illustration, "like marbles in a bag, they touch each other without sticking together." Of the two cases before us the first gives bad facts, but good reasoning; the latter bad reasoning, but good facts.

Such being the case it is clear that in any valid syllogism, it is a perfect matter of indifference what words we adopt for its several terms. We may adopt *any;* a fact which enables us to give the requisite amount of generality by using letters as symbols. Let Y stand for the middle term, Z for the major, and X for the minor.

The result will then be—

1.

Every Y is Z.
Every X is Y.
Every X is Z.

2.

No Y is Z.
Every X is Y.
No X is Z.

3.

Every Y is Z.
Some X is Y.
Some X is Z.

4.

No Y is Z.
Some X is Y.
Some X is not Z.

5.

Every Z is Y.
Some X is not Y.
Some X is not Z.

6.

Some Y is not Z.
All Y is X.
Some X is not Z.

7.

No Z is Y.
All X is Y.
No X is Z.

8.

Every Y is Z.
Some Y is X.
Some X is Z.

9.

No Z is Y.
Some X is Y.
Some X is not Z.

10.

No Y is Z.
Some Y is not X.
Some X is not Z.

Again, instead of—

| | | |
|---|---|---|
| Universal affirmative, | write | A. |
| Universal negative | ,, | E. |
| Particular affirmative | ,, | I. |
| Particular negative | ,, | O. |

Doing this, we may say that—

| | |
|---|---|
| The contradictories are | A and O. |
| The contraries are | A and E. |
| The subcontraries are | I and O. |
| The subalterns | A and I. |
| ,, | E and O. |

We may also say that such a syllogism as the first is A, A, A, in the first figure; such a one as the fifth A, E, E, in the third, and so on.

The memory may be helped. Combinations like A, A, A, A, E, E, and the like are not very easily remembered. Insert, however, consonants between them, and convert them into words. Let b*a*rb*a*r*a* be the name for syllogism 1:—c*e*l*a*r*e*nt that for syllogism 2, and so on whenever the vowels give the quality and quantity of the several propositions. This is actually done—the names being for,

1. B*a*rb*a*r*a*.

2. C*e*l*a*r*e*nt.

3. D*a*r*ii*.

4. F*e*r*io*.

5. B*a*r*o*k*o*.

6. B*o*k*a*rd*o*.

7. C*e*s*a*r*e*.

8. D*a*t*i*s*i*.

9. F*e*st*i*n*o*.

10. F*e*r*i*s*o*n.

It would be too much to say that the logic of even the ordinary syllogism ended here. There is much beyond the present details in the ordinary books. But some of this is unessential, some controversial; much historical, much (as has been already suggested) referable to language and the philosophy of classification and definition. Still there is some of it which is truly syllogistic. There are certain fallacies; there are certain compendious and elliptic modes of expression. The main details, however, are the few just given.

The logic just exhibited is the logic of three signs—*all, none,* and *some.* By excluding *none* we get a logic of *two: all* and *some.* This we arrive at by extending the criticism which applied to the particular to the *universal* negative. Let

*No man is perfect,*

be written

*All men are not perfect;*

and a new arrangement is the result. The negative is shifted, and all propositions are affirmative. There is a gain, here, on the side of generality. The details, however, of this have not been worked out.

Another logic of *two* signs is obtained by eliminating *all, i.e.,* by making it only a strong form of *some:* so that *none* and *some* (*not none*) are the only signs. What applies to the preceding applies here. For practical purposes *three* signs are convenient; for scientific purposes, they are sufficiently general. But are they not unnecessarily general? Cannot *more than three signs* be introduced with advantage on the side of convenience, and without loss in the way of generality? Lambert admitted the word *same* as a sign. Thus from

*Some men are philosophers:*
*The same are pagans;*

we infer,

*Some pagans are philosophers.*

What, however, if we admit as signs, the numerals 2, 3, 4, 50, and the like? What if we admit terms like *half,* or *more than half?* The syllogism thus formed has been called the numerically definite syllogism.

The details of this may be considered hereafter. The *object* of the present paper is to give the common syllogisms briefly.

